# Evaluating multiple criteria for species delimitation: an empirical example using Hawaiian palms (Arecaceae: *Pritchardia*)

**DOI:** 10.1186/1471-2148-12-23

**Published:** 2012-02-22

**Authors:** Christine D Bacon, Miles J McKenna, Mark P Simmons, Warren L Wagner

**Affiliations:** 1 Department of Biology, Colorado State University, Fort Collins, CO 80523-1878, USA; 2 Department of Botany, Smithsonian Institution, MRC-166, P.O. Box 37012, Washington, D.C. 20013-7012, USA; 3 Smithsonian Tropical Research, Box 0843-03092, Balboa, Ancón, Republic of Panamá

**Keywords:** Hawaii, Hybridization, Lineage sorting, Microsatellite, *Pritchardia*, Radiation

## Abstract

**Background:**

Robust species delimitations are fundamental for conservation, evolutionary, and systematic studies, but they can be difficult to estimate, particularly in rapid and recent radiations. The consensus that species concepts aim to identify evolutionarily distinct lineages is clear, but the criteria used to distinguish evolutionary lineages differ based on the perceived importance of the various characteristics of evolving populations. We examined three different species-delimitation criteria (monophyly, absence of genetic intermediates, and diagnosability) to determine whether currently recognized species of Hawaiian *Pritchardia *are distinct lineages.

**Results:**

Data from plastid and nuclear genes, microsatellite loci, and morphological characters resulted in various levels of lineage subdivision that were likely caused by differing evolutionary rates between data sources. Additionally, taxonomic entities may be confounded because of the effects of incomplete lineage sorting and/or gene flow. A coalescent species tree was largely congruent with the simultaneous analysis, consistent with the idea that incomplete lineage sorting did not mislead our results. Furthermore, gene flow among populations of sympatric lineages likely explains the admixture and lack of resolution between those groups.

**Conclusions:**

Delimiting Hawaiian *Pritchardia *species remains difficult but the ability to understand the influence of the evolutionary processes of incomplete lineage sorting and hybridization allow for mechanisms driving species diversity to be inferred. These processes likely extend to speciation in other Hawaiian angiosperm groups and the biota in general and must be explicitly accounted for in species delimitation.

## Background

Species are a fundamental unit in biological studies and their robust delimitation is essential to many fields of evolutionary biology, particularly systematics, biogeography, and conservation biology. Lineage separation and divergence form a temporal process that may render populations monophyletic, reproductively isolated, ecologically divergent, and/or morphologically distinctive. These properties serve as operational criteria for systematists to delimit species and they can occur at different times or orders during speciation. De Queiroz [1,2] proposed that at the root of all modern species concepts is the general agreement on the fundamental nature of species: species are separately evolving metapopulation lineages. The perspective that species are lineages, and that multiple criteria should be used to identify them, has been termed the general lineage species concept [1]. Applying this lineage-based framework to species delimitation shifts the focus from a single operational criterion and increases the importance of sampling multiple lines of evidence. Species delimitation is notoriously difficult when alternative criteria delimit incongruent species boundaries, but this is to be expected in recent radiations (e.g. [3-5]). Evaluating multiple criteria not only increases our ability to detect recently separated lineages, but also can provide stronger support for lineage separation when they are in agreement [2,6,7].

The difficulty in recognizing species and their limits (the "species problem" [8]) is particularly compounded on islands. Because most islands are considerably younger terrestrial systems than continental areas [9], there has generally been less time for the completion of speciation processes. Time is an important factor for incomplete lineage sorting because the existence of ancestral polymorphism and differential extinction thereof can cause bias in phylogenetic inference (e.g. [10]) and the identification of distinct lineages (e.g. [11]). Furthermore, the tendency for island colonizers to quickly fill available habitat often leads to species that are ecologically isolated but not considerably diverged genetically, potentially leading to hybridization if mating barriers are broken down due to secondary contact (e.g. [12,13]). The evolutionary processes of incomplete lineage sorting and hybridization cause the "species problem" to be compounded on young, volcanic islands. Hawai'i is the longest archipelago on earth and has developed linearly in a sequential fashion from a volcanic hotspot [14]. Recent study of the extant high islands has shown that the terrestrial biota evolved over the last 29-23 Ma [15] and that they harbor the highest degree of endemism of any known flora [16,17]. The species richness of the Hawaiian Islands also contributes to the Polynesian/Micronesia biodiversity hotspot [18]. Difficulties in delimiting species is not restricted to angiosperms on the Hawaiian Islands (e.g. [19-24]), but has also been highlighted in *Hylaeus *bee [25] and spoon tarsus *Drosophila *[26] studies.

**Figure 1:**
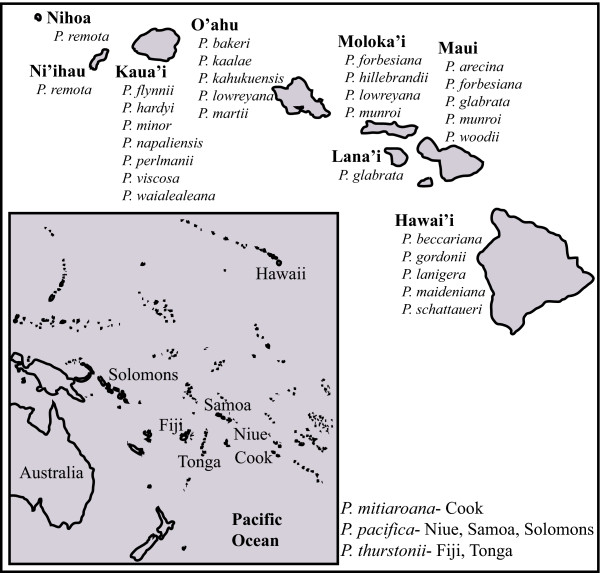
The geographic distribution of Hawaiian and eastern Pacific *Pritchardia* species according to the most recent morphological classification [29,30].

An excellent group within which to address the evaluation of species boundaries based on various delimitation criteria is the Hawaiian *Pritchardia *(Arecaceae/Palmae) radiation. *Pritchardia *is economically important as a widely cultivated ornamental palm [27], displays high endemism, and is a conservation priority for the State of Hawaii (15 threatened or endangered species [28]). *Pritchardia *is one of the most species-rich plant genera in Hawaii [29] and contains 27 currently recognized, primarily single-island endemic species (Figure 1, [29,30]). The genus also occurs on small islands in the eastern Pacific (Cook, Fiji, Niue, Samoa, Solomon, Tonga). Based on the most recent phylogenetic results *Pritchardia *is monophyletic and sister to *Copernicia*, although definitive generic relationships among *Copernicia, Pritchardia*, and *Washingtonia *were uncertain due to gene-tree incongruence [31]. Previous work has also shown that the North American and Caribbean lineage leading to *Pritchardia *colonized the eastern Pacific and then dispersed to Hawaii between 3.5-8 million years ago (MA; mean stem-crown ages [31]). Although no explicit species concept was applied, Hodel [29] recently revised *Pritchardia *using morphological data. Hodel [29] noted that character states were often difficult to define because *Pritchardia *morphology is highly labile based on environmental conditions (see also [32,33]). Accurate estimation of species limits is important to understanding the evolution and radiation of *Pritchardia *species and is essential to conservation efforts on the Hawaiian Islands.

Species concepts can address both the evolutionary patterns consistent with evolution along lineages and the evolutionary processes that are fundamental in maintaining distinct lineages (e.g. [7]). Under the phylogenetic species concept I (PSCI [34]), species are defined as "the smallest aggregation of populations (sexual) or lineages (asexual) diagnosable by a unique combination of character states in comparable individuals" (p. 211 [35]). To apply PSCI, fixed (or mutually exclusive) character-state differences are used as evidence to infer that gene flow has ceased between the sampled populations in population aggregation analysis (PAA [36]). An alternate version of the phylogenetic species concept (PSCII) requires exclusivity to recognize a species and differs from PSCI by basing species recognition strictly on monophyletic groups ([37]; properly exclusive lineages [38]). A third alternative is the genotypic cluster species concept (GSC [39]), which defines species as genetic groups with few or no intermediates between them. The GSC can be implemented using a variety of clustering algorithms or assignment tests. Looking across species delimitation criteria allows for the implementation of the general-lineage species concept where the greater the number of criteria satisfied by a putative lineage, the more likely it is to represent an independent evolutionary trajectory [2].

Adaptive radiations are difficult evolutionary scenarios to evaluate because phylogenetic lineages may be so recently separated that each species' alleles have not coalesced since the time of speciation [40]. Among recently diverged species, genealogies inferred from independent genomic regions are likely to disagree due to the differential sorting of ancestral polymorphism into daughter lineages such that each inferred gene tree might differ from the species tree (e.g. [41]). Because estimation of a coalescent species tree explicitly models incomplete lineage sorting, its comparison with the simultaneous-analysis [42,43] allows for the inference of hybridization from any incongruence between the two topologies when only orthologous alleles are sampled.

In this study we aim to provide a comprehensive assessment of species diversity in *Pritchardia *using a multifaceted approach and independent sources of plastid, nuclear, and morphological data to assess three species-delimitation criteria - monophyly, the absence of genotypic intermediates, and diagnosability using mutually exclusive character states. We test whether currently recognized *Pritchardia *species merit taxonomic recognition as distinct evolutionary lineages, particularly with respect to the accumulation of evidence in favor of their delimitation. We also take advantage of the power of the coalescent to infer the species tree to understand potential conflicts in our results that can be introduced by incomplete lineage sorting and/or hybridization.

## Results

**Figure 2:**
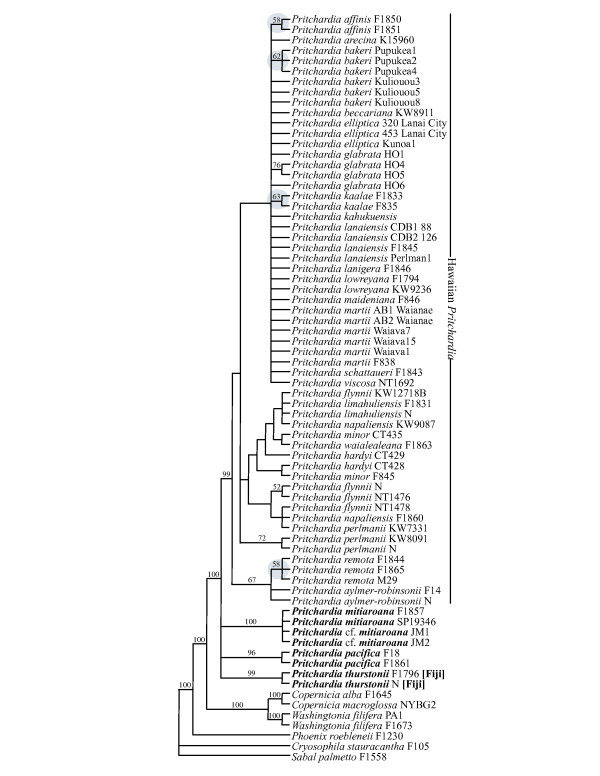
**Analysis 1 (A1) parsimony tree inferred from DNA sequence and nuclear microsatellite data with which the phylogenetic species concept II was applied**. Currently recognized species that are supported in this analysis are indicated with a grey circle; species from the eastern Pacific are in bold font; and the Fijian species is also indicated.

Gene-tree incongruence was detected among five of the seven loci for the resolution of the sister group of *Pritchardia *and among two of the seven loci for the sister group of Hawaiian *Pritchardia *(Additional file 1). The analysis 1 (A1) dataset comprised seven genes and five microsatellite loci for 72 individuals; 134 characters are variable and 81 are parsimony-informative within *Pritchardia *(Figure 2). Application of PSCII to the *Pritchardia *relationships in our A1 matrix indicated that the three currently recognized species of eastern Pacific *Pritchardia *(*P. thurstonii, P. pacifica*, and *P. mitiaroana*; Figure 2) are each distinct evolutionary lineages. Despite low branch support, Hawaiian *P. affinis, P. kaalae*, and *P. remota *were resolved as unique monophyletic groups and satisfy the PSCII criterion. A monophyletic group of *P. bakeri *from Pupukea, O'ahu was also resolved and likely represents population structure within the Ko'olau mountain range. A clade that included a subset of *P. glabrata *individuals and another clade that included a subset of *P. perlmanii *individuals were resolved, consistent with each of these being distinct evolutionary lineages according to the PSCII criterion.

**Figure 3:**
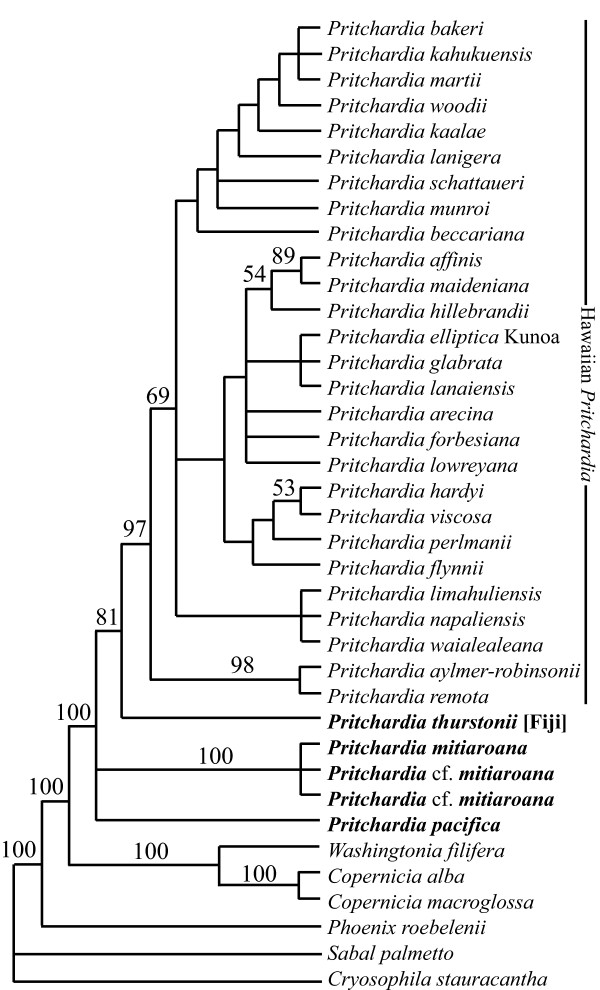
**Analysis 2 (A2) parsimony tree inferred from composite taxa constructed from the data in A1 together with isozyme and morphological data and showing inter-specific relationships where eastern Pacific species are indicated with bold font and the Fijian species is also indicated**.

The 35 *Pritchardia *species sampled in the analysis 2 (A2) matrix was reduced to 32 by deleting wildcard taxa identified from comparisons of the Adams and strict consensus trees (Figure 3). The reduced A2 matrix has 79 parsimony informative characters. The eastern Pacific *Pritchardia *species *P. pacifica *and *P. mitiaroana *were resolved as part of a basal polytomy within *Pritchardia*, but there was strong support for monophyly of *P. mitiaroana *[100% jackknife support (JK)]. *Pritchardia thurstonii *was well supported (81% JK) as the sister species to the Hawaiian clade, which was strongly supported (97% JK) as a monophyletic group. *Pritchardia aylmer-robinsonii *and *P. remota *were strongly supported as sister species (98% JK), consistent with their synonymy. *Pritchardia affinis *and *P. maideniana *were well supported (89% JK) as sister taxa, also consistent with recent synonymy, and *P. hillebrandii *was weakly supported (54% JK) as its sister species. *Pritchardia hardyi *and *P. viscosa *were also weakly supported (53% JK) as sister species.

**Figure 4:**
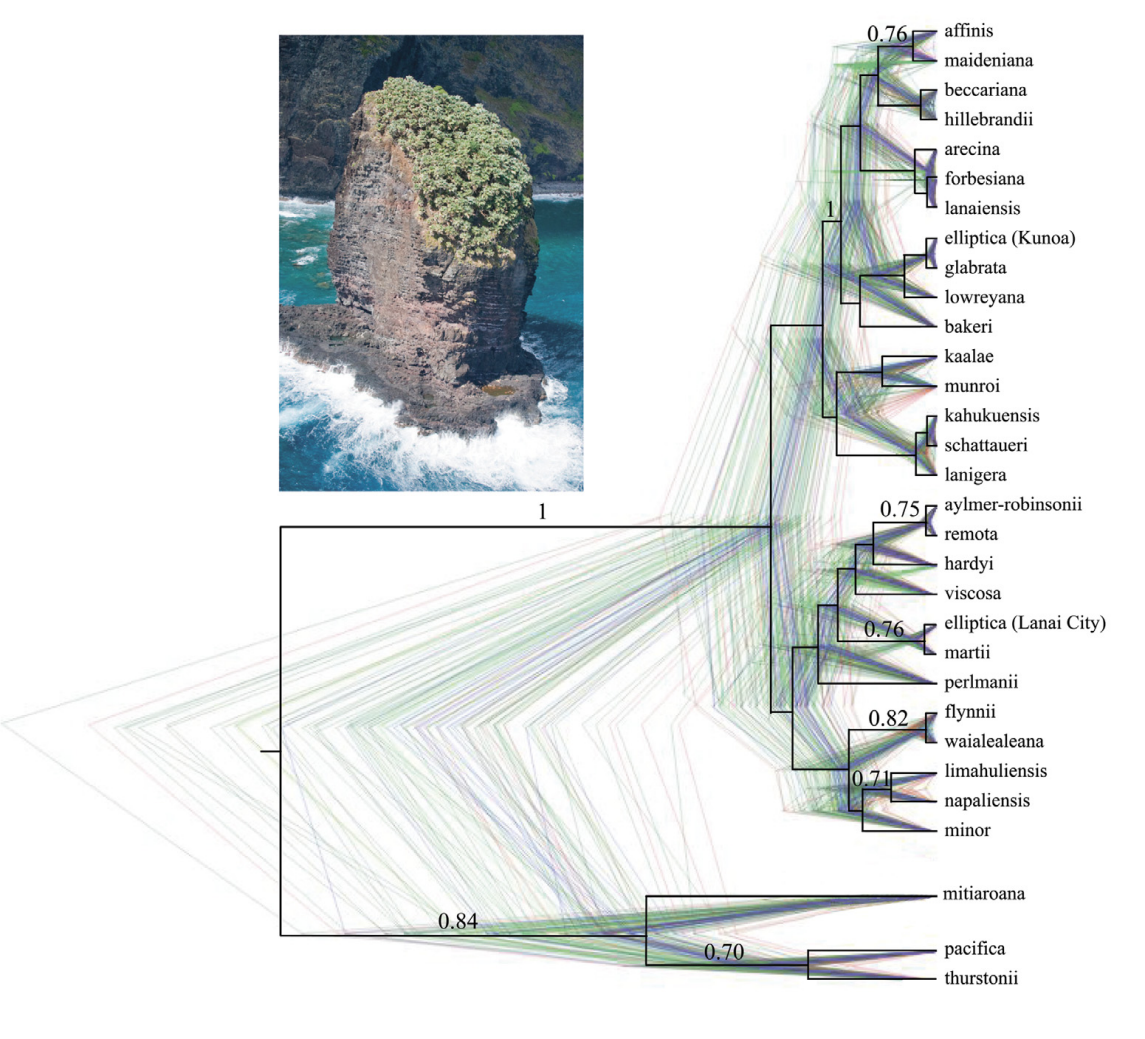
Relationships amongst predefined *Pritchardia *lineages where resampled posterior species trees as inferred from *BEAST are in color and posterior probabilities ≥0.5 based on the single combined tree are overlaid in black. *Pritchardia hillebrandii*, which has one of the most restricted distributions in the genus, is pictured on Huelo Islet (photo and copyright D.R. Hodel).

We explicitly modeled incomplete lineage sorting through the use of a multispecies coalescent tree for the sequence data (Figure 4). The topology did not have any mutually well-supported (≥75% branch support) conflicts with the A1 or A2 trees. The congruence between methods indicates that the trees used for species delimitation (A1) and for inference of inter-specific relationships (A2) is not biased by patterns of lineage sorting. The *BEAST species tree resolved four moderately supported groupings of Hawaiian individuals not seen in the comparable A2 analysis (Figure 3), although this may be due to inherent differences between parsimony and Bayesian tree reconstruction and JK support vs. posterior probabilities [44,45]. The recently synonymized *P. affinis *into *P. maideniana, P. aylmer-robinsonii *into *P. remota*, and *P. limahuliensis *into *P. napaliensis *[29] were each resolved as monophyletic groups. Although the posterior probabilities for these cases of synonymy were modest [between 0.71 and 0.76 posterior probability (PP)], these three taxonomic changes based on morphology [29] are consistent with our molecular results.

**Figure 5:**
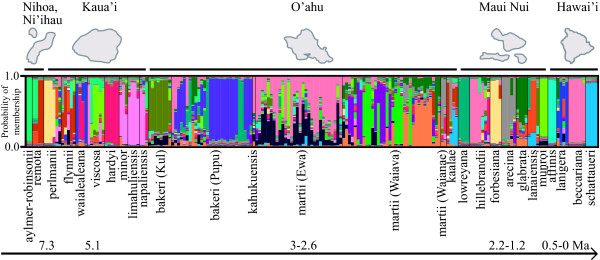
**Putative species are labeled below and Hawaiian distributions from the oldest to the youngest island are indicated above the bar plot. *Pritchardia bakeri* populations from Kuliouou and Pupukea are abbreviated as Kul and Pupu respectively. **Evidence for distinct evolutionary lineages without significant admixture or the presence of intermediates supports *Pritchardia affinis, P. aylmer-robinsonii, P. hardyi, P. munroi*, and *P. schattaueri *as independent lineages. Putative species are labeled below and Hawaiian distributions from the oldest to the youngest island are indicated above the bar plot.

Significant p-values indicating disequilibrium were detected in the '90' microsatellite locus for two populations (*P. martii *Waianae and *P. lanigera *2) and no evidence for stutter, large allele dropout, or null alleles was detected at any of the loci based on 99% confidence intervals. Structure analyses resulted in mean LnP(*K*) values that appeared to plateau when graphed in Structure Harvester, making it difficult to identify the most likely *K *value for the number of genetic groups present in the data. Therefore the Δ*K *method was applied and the highest probability for the number of groups that individuals were assigned to (*K*) was 21 (Mean LnP(*K*) = -2802, Δ*K *= 3.84). Upon visualization of population assignments from across the Structure iterations, the presence of genetic intermediates between *Pritchardia *species was evident (Figure 5). Levels of admixture were particularly high in areas of sympatry such as in the Makaleha and Namolokama ranges in Kaua'i where up to five species overlap in geographic distribution [*P. flynii, P. hardyi, P. perlmanii *(albeit to a lesser extent), *P. viscosa*, and *P. waialealeana*] and in the Ko'olau Mountains of O'ahu where three species are sympatric (*P. bakeri, P. kahukuensis*, and *P. martii*). Genetic subdivision and little admixture between species were detected among *P. affinis, P. aylmer-robinsonii, P. beccariana, P. forbesiana, P. hardyi, P. lowreyana, P. munroi*, and *P. schattaueri *and these eight groups meet the necessary criterion for species delimitation according to the GSC of high probability of assignment to their respective genetic groups (> 0.8 membership coefficient). However, the individual Q matrix of assignment to groups shows that *P. beccariana, P. forbesiana*, and *P. lowreyana *do not represent distinct evolutionary lineages according to the GSC because they do not group as unique clusters; with other individuals in the Q matrix having > 0.8 PP of falling within those groups. Although the 0.8 cut-off is arbitrarily defined, the maximum values from the Q matrices show a discontinuous distribution where individuals have a membership coefficient of > 0.8, while the remaining have < 0.5 with few in between. Therefore, based on our data, only *P. affinis, P. aylmer-robinsonii, P. hardyi, P. munroi*, and *P. schattaueri *meet the necessary and sufficient criteria as distinct evolutionary lineages without intermediates according to the GSC (Table 1).

**Table 1:** Conformance of currently recognized Hawaiian *Pritchardia *with three distinct criteria for species delimitation

Hawaiian *Pritchardia*	Monophyletic	Genotypic cluster	Diagnosable
*affinis*	**58%**	**12; 0.91**	No
*arecina*	No	19; 0.71	**Yes**
*aylmer-robinsonii*	No	**18; 0.91**	**Yes**
*bakeri *Kuliouou	No	13; 0.67	No
*bakeri *Pupukea	**62%**	17; 0.51	**Yes**
*beccariana*	No	14; 0.90	No
*elliptica *Kunoa	No	4; 0.48	No
*elliptica *Lanai City	No	-	No
*flynii*	No	11; 0.40	No
*forbesiana*	No	10; 0.83	**Yes**
*glabrata*	**76%**	20; 0.58	**Yes**
*gordonii*	-	**-**	**Yes**
*hardyi*	No	**7; 0.86**	**Yes**
*hillebrandii*	No	14; 0.59	**Yes**
*kaalae*	**63%**	11; 0.31	**Yes**
*kahukuensis*	No	21; 0.52	No
*lanaiensis*	No	8; 0.46	No
*lanigera*	No	9; 0.55	No
*limahuliensis*	No	4; 0.78	No
*lowreyana*	No	15; 0.89	**Yes**
*maideniana*	No	-	**Yes**
*martii *Ewa	No	21; 0.44	No
*martii *Waiawa	No	19; 0.54	**Yes**
*martii *Waianae	No	11; 0.31	No
*minor*	No	4; 0.62	No
*munroi*	No	**16; 0.83**	**Yes**
*napaliensis*	No	4; 0.52	No
*perlmanii*	**72%**	10; 0.57	No
*remota*	**58%**	11; 0.51	**Yes**
*schattaueri*	No	**2; 0.91**	**Yes**
*viscosa*	No	16; 0.40	**Yes**
*waialealeana*	No	9; 0.50	**Yes**
*woodii*	-	-	**Yes**

Monophyly, as required by the phylogenetic species concept II, is shown as parsimony jackknife branch support. Genotypic clusters are labeled with their inferred genetic group and their estimated membership coefficient. Diagnosability to satisfy the phylogenetic species concept I was determined by PAA

Four distinct lineages were identified within *Pritchardia *microsatellite data using PAA, 33 in the sequence data and 12 in morphology, although individuals with missing data for diagnostic characters were left out of aggregations to avoid collapsing otherwise distinct groups (Additional file 2). For example, in the sequence data seven terminals had missing data for diagnostic characters and were arbitrarily assigned to a single group rather than collapsing the otherwise diagnosable groups. Due to differential sampling only the individuals sampled for the sequence dataset were used to perform PAA across the microsatellite and morphological data. In the three datasets we generated for this study, 43 lineages were indentified that are diagnosable and satisfy the PSCI. Of the 43 PSCI species, unique combinations of character states support 18 currently recognized *Pritchardia *species (Table 1).

## Discussion

The Hawaiian Islands have an unparalleled number of well-studied examples of adaptive evolution because of their high ecological heterogeneity, volcanic origin, and isolation from the nearest continental land mass [46]. Despite the limited time available for diversification in comparison to ancient landmasses [15,47], the Hawaiian Islands have the highest degree of endemism of any known flora [16,32]. Within the Hawaiian Islands, many angiosperms show evidence for recent and rapid radiations, which frequently make species delimitation difficult (e.g. [21-26]). We applied three species-delimitation criteria to identify evolutionary lineages in Hawaiian *Pritchardia*. Robust species delimitations are important for *Pritchardia *because many of the currently recognized species are of conservation concern and threats continue to increase due habitat degradation and invasive herbivores and competitors [48].

We applied the criterion of monophyly to test whether currently recognized *Pritchardia *species are distinct evolutionary lineages using PSCII. Maximum parsimony (MP) analysis of the A1 matrix revealed support for *P. affinis, P. glabrata, P. kaalae, P. perlmanii *and *P. remota *as clades (Figure 2). Although these are weakly supported lineages, they satisfy the monophyly requirement of PSCII [37]. Despite its popularity, monophyly as inferred from a phylogenetic tree may be a poor indicator of whether evolutionary lineages are distinct in the presence of gene flow [49,50] or due to the error associated with randomly sampling few individuals from a complex underlying genealogy [51]. Furthermore, decoupling hybridization from incomplete lineage sorting on a phylogeny is difficult in recently diverged species because both produce the same pattern of few to no polymorphisms between morphologically identifiable species [52-54].

The genotypic cluster criterion defines species as "distinguishable groups of individuals that have few or no intermediates when in contact" (p. 296 [39]). A Bayesian assignment test was used to quantify the degree of admixture (essentially the absence of intermediates) between species. Although issues can arise with imperfect geographical sampling, especially in cases of isolation by distance or environmental gradients (e.g. [55]), strong signal for the delimitation of *P. affinis, P. aylmer-robinsonii, P. hardyi, P. munroi*, and *P. schattaueri *was detected with high probability of assignment to unique populations. A lack of intermediates satisfies this species criterion and these five groups are distinct evolutionary lineages according to the GSC. On the other end of the speciation spectrum, sympatric species appear to have ongoing gene flow among lineages where the probabilities of membership among some heterogeneous individuals and populations were shared (Figure 5), particularly in the mountains of Kaua'i and O'ahu.

Under the criterion of diagnosability, species are identified as the smallest aggregation of populations diagnosable by a unique combination of character states [35]. Using PAA, 43 lineages were identified as diagnosable and although they conform to PSCI as independent lineages, we do not advocate their formal recognition as species. Rather, our goal was to implement the general lineage species concept using multiple species-delimitation criteria to reach a more stable taxonomic solution for the Hawaiian *Pritchardia*. Furthermore, PAA can be highly sensitive where incomplete sampling of characters, individuals within populations, or populations can each lead to incorrect assessment of species [36]. In our Hawaiian *Pritchardia *data, the nucleotide sequence matrix had 24% percent missing or ambiguous data, which was mostly due to a lack of sampling in the Malate synthase (MS) gene (Additional file 1). The microsatellite and morphological matrices had only 0.05% missing data. Between one and six individuals were sampled per population with an average of 1.5 individuals and 1.7 populations per species in the nucleotide sequences of the A1 matrix. Between one and 34 individuals were sampled per population with an average of seven individuals and two populations per species for the microsatellite matrix. The morphological matrix comprised character states that were fixed within currently recognized species and were not typically scored from the actual specimens used in the sequence-based and microsatellite analyses. Additionally, ten of the morphological characters were derived from species descriptions [29,30] rather than herbarium material. Certainly no study is immune to these types of weakness, but we recognize that undersampling of individuals within populations and populations within species have affected the PAA results in this study by over-splitting and thus increasing the number of apparent species.

### Distinct evolutionary lineages of *Pritchardia*

As currently defined, *Pritchardia *species are primarily recognized by their geographic distributions and a suite of morphological characters [29,30]. Yet when considering distinct evolutionary lineages identified in this study, none of the *Pritchardia *species satisfy all the species-delimitation criteria that we applied. Some species-delimitation criteria recognize more lineages than others in part because criteria are met at different times during cladogenesis [2]. Furthermore, when considering the amount of data used in the application of each criterion to infer species delimitations in Hawaiian *Pritchardia *we found the method that uses the most data, PAA, was the most powerful because it recognized the greatest number of splits.

Seven *Pritchardia *lineages satisfy two species-delimitation criteria (*P. affinis, P. glabrata, P. hardyi, P. kaalae, P. munroi, P. remota*, and *P. schattaueri*). The taxonomic status of *P. affinis *and *P. remota *are discussed in the interpretation of the A2 and coalescent species trees (see below). *Pritchardia lanaiensis *and *P. elliptica *were recently synonymized into *P. glabrata *[29], yet our results are inconsistent with this designation because of the diagnostic grouping of all *P. glabrata *sensu stricto individuals in PAA (Additional file 2).

*Pritchardia hardyi, P. munroi*, and *P. schattaueri *are all distinct lineages based on the species-delimitation criteria of a lack of intermediates and the presence of diagnostic character states. These results are consistent with Hodel's [29] description of morphological autapomorphies that define each of these three independent lineages. *Pritchardia kaalae *is identified as an independent lineage based on the formation of a monophyletic group and the presence of diagnostic character states. Despite its distinction as an independent lineage, *P. kaalae *appears to have significant levels of admixture based on the Structure results, particularly with Waianae and central Ko'olau (Waiava) populations of *P. martii *(Figure 5). Admixture may be indicative of the *Pritchardia*-dominated ancestral forest of the extensive O'ahu plain that spanned the Waianae and Ko'olau mountains and facilitated gene flow between ranges [46,56]. The once-contiguous palm forest likely formed an isolation-by-distance-based cline of gene flow, and extinction of the intervening lowland populations may have subsequently formed reproductively isolated lineages.

Eleven *Pritchardia *lineages satisfy only one species-delimitation criterion (*P. arecina, P. forbesiana, P. gordonii, P. hillebrandii, P. lowreyana, P. maideniana, P. perlmanii, P. viscosa, P. waialealeana*, and *P woodii*). Some of these *Pritchardia *lineages may be recognized as independent due to the sampling artifacts described above. This is particularly a concern with *P. gordonii *and *P. woodii*, which were only sampled for morphology, and *P. hillebrandii*, which was not sampled for the sequence data. Future efforts to tease apart distinct evolutionary lineages in *Pritchardia *should focus on these particular groups, as well as areas of sympatry, with increased sampling of both individuals within populations and of populations within species.

### Sister-group and inter-specific relationships of *Pritchardia*

In previous studies the sister group of *Pritchardia *has been inferred to be either *Copernicia *(53% bootstrap, BS, in maximum representation with parsimony analysis [57]; < 50% JK/BS and 0.89 PP [31]) or *Washingtonia *(52% BS [58]). Our study is consistent with previous work showing the close relationships among the three genera (*Copernicia, Pritchardia*, and *Washingtonia*). In the A1 and A2 matrices, *Copernicia *and *Washingtonia *together are inferred to be the sister group to *Pritchardia *with strong support (100% JK; Figures 2 and 3).

We formed composite terminals [59] from the A1 matrix where taxa were combined at a level for which monophyly is assumed a prior thereby reducing missing data. Sequence data was augmented with allozyme and morphological data to construct the A2 matrix for simultaneous analysis of inter-specific relationships (Figure 3). The A2 matrix did not incorporate potential hybrid lineages because of the terminal omission iterations and was compared to the coalescent species tree to assess effects of incomplete lineage sorting. In the A2 tree, *P. thurstonii *is sister to the Hawaiian clade, which is well supported as monophyletic (97% JK) and consistent with Bacon et al. (64% BS/65% JK [31]). Zielger [60] proposed the sister relationship between Fijian and Hawaiian *Pritchardia *based on his hypothesis of an adaptive shift in fruit size upon colonization of the Hawaiian Islands. The sister relationship between Fijian and Hawaiian angiosperms has also been noted in *Cyrtandra *[23] and *Pittosporum *[21,26], but not in taxa that ultimately descended from American ancestors [61], such as *Pritchardia*.

The strongly supported sister relationship *P. aylmer-robinsonii *and *P. remota *(98% JK, Figure 3) is consistent with their synonymy [29]. Excluding *P. remota *from Nihoa and Ni'ihau, the backbone of the Hawaiian clade is a trichotomy. Weak support was provided for a sister relationship between *P. hardyi *and *P. viscosa *(53% JK), which had been previously suggested based on their flat leaf blades, the density of lepidia on the abaxial surface of the leaf, and their stiff leaf tips [29]. Hodel [29] also identified a close relationship between *P. maideniana *(including *P. affinis*) and *P. hillebrandii *based on morphological aspects of the lepidia and inflorescences, for which we inferred a well-supported *P. maideniana *sensu lato (89% JK) that was weakly supported as sister to *P. hillebrandii *(54% JK).

The coalescent-species-tree approach has been suggested to be a more accurate estimation of lineage splitting than concatenation because it can model the stochastic forces that drive population divergence [40,62-64]. Yet missing data and other issues with species-tree estimation such as mutational and coalescent variance can have detrimental effects on modeling incomplete lineage sorting (e.g. [65]). Another important consideration with species-tree estimation is that species are defined a priori and the coalescent model assumes species are monophyletic. This can be highly unlikely in recent radiations where ancestral species are still extant. Despite these issues, the advantage of directly modeling intraspecies polymorphism and incomplete lineage sorting makes species-tree estimation an important approach to data exploration in the identification of evolutionary lineages, especially in rapid species radiations [62].

The coalescent-species-tree topology provided moderate branch support for three clades that are consistent with recent synonymy [*P. aylmer-robinsonii *(0.75 PP), *P. maideniana *(0.76 PP), and *P. napaliensis *(0.71 PP); Figure 4]. The species tree identified *P. flynnii *and *P. waialealeana *as sister taxa, which together are sister to *P. minor *(Figure 4). Lastly, individuals planted by early Hawaiian naturalist George Munro in Lana'i City, Lana'i had been hypothesized to represent the extinct *P. elliptica *lineage (R.W. Hobdy, pers. comm. 2008), but are here shown to be consistent with a *P. marti *source from O'ahu (0.76 PP; see also Additional file 3) and separated from *P. elliptica *individuals collected from natural populations in Kunoa Valley by eight branches, one of which is highly supported (1.0 PP; Figure 4). Secondly, the species tree was used to test for congruence with the simultaneous-analysis A2 topology. Because the two distinct methods generally resolved the same well-supported clades, we can infer that the extrapolation from the gene trees to the phylogenetic tree is likely accurate in the simultaneous analysis. This is not to say that the process of lineage sorting has not occurred, but rather we have no evidence that it has confounded the species-level relationships inferred from the simultaneous-analysis tree.

### Incomplete lineage sorting, the tempo of radiation, and hybridization in *Pritchardia*

The identification of distinct evolutionary lineages is a necessary precursor to the delimitation of species [2]. Satisfaction of multiple species criteria can ensure accurate, stable, and uncontroversial species delimitations (e.g., [4,7]). For taxa of conservation concern, accurate identification of lineages may facilitate management efforts by focusing on distinct species, rather than ambiguous groups. Our results, which are based on data from both the plastid and nuclear genomes, show little sequence differentiation among most *Pritchardia *species. The lack of differentiation may be due to incomplete lineage sorting, the tempo of the *Pritchardia *radiation, and/or hybridization between sympatric species, and distinguishing between these factors can be difficult.

Incomplete lineage sorting is one hypothesis for gene-tree incongruence and a lack of resolution within island radiations. Differential lineage sorting can bias species inference and may be further compounded by the estimated long generation time for other tropical understory palms that have undergone island colonization (e.g., 68-year mean in the Fijian endemic *Balaka microcarpa *[66]). Large ancestral effective population sizes have been hypothesized from fossil evidence and *Pritchardia *has been shown to be the dominant component of pre-human Quaternary forests on the Hawaiian Islands (2.6 Ma-822 year before present [67,68]). Despite this, coalescence times for Hawaiian *Pritchardia *species are likely to be shorter than their continental tribal counterparts. Congruence between the simultaneous and species-tree analyses together with information on coalescence times suggests that differential lineage sorting did not drive current diversity patterns within *Pritchardia*.

A general trend emerging from this and other phylogenetic studies on the Hawaiian flora is the difficulty in estimating relationships among woody and long-lived groups [e.g., *Cyrtandra *[19], lobeliads (e.g. [69]), *Melicope *[20]; *Metrosideros *[21,70]; *Pittosporum *[22,23]; *Pyschotria *[71]; *Santalum *[24], *Schiedea *[5], and the silversword alliance (e.g. [72])]. Another example is Hawaiian *Pritchardia*. Aside from the sympatric species, the lack of resolution may be caused by the insufficient time for divergence between lineages. Because of the age of the oldest extant Hawaiian Island (Nihoa; 7.3 Ma [14,15,47]) and because the *Pritchardia *colonization of the Hawaiian Islands was estimated to occur between 3.5-8 Ma (mean stem to crown-stem ages [31]), an average of three new species would have had to form every million years to account for the 24 currently recognized species in the radiation. Clearly this rapid rate of cladogenesis has not allowed for much divergence within the Hawaiian *Pritchardia *radiation.

We also suggest that hybridization has played a key role in the diversification of Hawaiian *Pritchardia *lineages from geographic regions of sympatry of Kaua'i (*P. flynnii, P. limahuliensis, P. minor, P. napaliensis, P. waialealeana*, and *P. viscosa*) and O'ahu (*P. bakeri, P. kahukuensis*, and *P. martii*). Removing wildcard terminals through the use of Adams consensus trees may be biased towards deletion of hybrids given that they are expected to be resolved as basal lineages [73] and our iterative exclusion process is consistent with the exclusion of hybrids because 66% (22 of the 33) of the excluded terminals were from areas of high sympatry such as in the Makaleha and Namolokamain ranges in Kaua'i and in the Ko'olau Mountains of O'ahu. Examination of the character conflict present was hampered by a general lack of resolution in the gene trees (each nDNA versus the single cpDNA tree; Additional files 4 and 5). Despite this, review of the parsimony-informative sequence characters revealed six polymorphisms found on both forward and reverse sequence reads that suggest introgression in two genes given how different the alleles are (nuclear MS amongst *P. perlmanii *individuals and plastid *trnD-trnT *amongst *P. hardyi *individuals). Although widespread hybridization has been observed in cultivation [74,75], it has been difficult to detect in the field due to the high phenotypic plasticity that characterizes *Pritchardia *[29,32].

## Conclusions

The ability to hybridize is common among island species (e.g. [9]) and has likely been a major force in shaping other Hawaiian angiosperm lineages such as *Metrosideros *[21,70], *Pittosporum *[22,23], and silverswords (e.g. [76]). Outside of the lack of reproductive barriers or incompatibility mechanisms, anthropogenic change on the archipelago may have caused a breakdown of species boundaries. For example, native Hawaiians cultivated *Pritchardia *species in coastal settlements and although they had a variety of ethnobotanical uses (reviewed in [77]), the leaves and fibers were primarily used for thatching. The movement of plants by humans could have introduced new genotypes into existing coastal native species and admixed with other cultivated species. Also, the likely extinction of natural pollinators and dispersers and the introduction of invasive species that generally have higher mobility and efficiency [78] may also facilitate gene flow between populations and species.

Research at the interface of population genetics and phylogenetics is greatly expanding, as seen in the increasing numbers of publications on coalescent methods to infer species trees (e.g. [40,62-64]). A limitation to the current implementations of species-tree methods is the assumption of lack of gene flow among lineages, yet in empirical studies this assumption is often violated, especially at the taxonomic level these methods are designed for. Although there are methods that model gene flow as well as the coalescent (i.e., the isolation-with-migration model of Hey & Nielsen [79] or the hierarchical approximate Bayesian computation approach of Huang et al. [80]), these approaches do not provide an estimate of a species tree under a model of divergence with gene flow and may be less powerful than species-tree estimates because they require such strong priors (e.g., on migration rates [62]). To best address species delimitation in rapid radiations, especially in island groups like *Pritchardia *palms, methods that allow for simultaneously capturing vertical and horizontal inheritance of genetic information are needed, but are not yet available ([81] but see [82]).

## Methods

### Phylogenetic analyses

Total genomic DNA was extracted from silica-gel dried leaves following Alexander et al. [83]. Sequences for three plastid (*matK, ndhF*, and *trnD-trnT*) and four nuclear loci (CISPs 4 and 5, MS, and RPB2) were generated ([84-88] respectively). Amplified products were purified using Qiagen PCR purification kits and sequenced by the Cancer Research Center DNA Sequencing Facility at the University of Chicago or at Macrogen. All 502 new sequences generated in this study have been deposited in GenBank under accession numbers JF904936 to JF905438 (Appendix A).

Two phylogenetic analyses were conducted within *Pritchardia*. A1 included sequence data generated from seven loci and microsatellite data coded as multistate characters with heterozygous individuals coded as subset polymorphisms. Sampling for A1 included all previously recognized *Pritchardia *species except for *P. gordonii *and *P. woodii*, which are both recently described species with highly restricted distributions and are considered endangered [29]. Based on a recent tribal-level analysis [31] two species of each of the most closely related genera (*Copernicia *and *Washingtonia*) and three other Coryphoideae (*Cryosophila, Phoenix*, and *Sabal*) were sampled as outgroups. The initial simultaneous analysis included 105 terminals.

Preliminary nucleotide alignments were obtained independently for each of the seven loci using default parameters in MUSCLE v3.6 [89] and manual adjustments were performed in MacClade v4.03 [90] following Simmons [91]. Each parsimony-informative character was confirmed by rechecking chromatograms in Aligner (CodonCode Corp., MA). MP tree searches were conducted using 1,000 random addition tree-bisection-reconnection (TBR) searches in PAUP* v4.0b10 [92] with a maximum of ten trees held per replicate. MP JK analyses [93] were conducted using PAUP* and 1,000 replicates were performed with 100 random addition TBR searches per replicate. Maximum likelihood (ML [94]) analyses of nucleotide and microsatellite characters from each of the molecular data matrices were performed. jModeltest v0.1.1 [95] was used to select the best-fit likelihood model for each data matrix using the Akaike Information Criterion [96] without considering invariant-site models following Yang [97]. Searches for optimal ML trees and 1,000 BS replicates [98] in the CIPRES Portal v2.2 used the RAxML-HPC2 algorithm [99,100]. Adams consensus trees [101] from parsimony analyses were examined using the A1 dataset to identify wildcard terminals [102] of uncertain phylogenetic position that were then omitted. Iterations were conducted until a trade-off was reached between sacrificing taxonomically important terminals and gaining resolution in the strict consensus tree. A total of 72 of the original 105 terminals were included in the final A1 matrix.

A2 incorporated the A1, morphological, and isozyme data and was reduced to 35 composite terminals representing all putative *Pritchardia *species. Nine discrete morphological characters of flower and fruit morphology were measured from specimens at BISH, NY, PTBG, and US and ten morphological characters were derived from species descriptions ([29,30] Table 2). To include lineages that are not currently recognized as species [29,30] morphological character states were extrapolated from recognized species to now synonymous entities. We did not incorporate the preliminary morphological matrix from Gemmill [77] because of scoring inconsistencies. A matrix of seven variable isozymes was derived from Gemmill [77]. Three terminals (*Pritchardia gordonii*, cultivated 'elliptica' from Lana'i City, Lana'i, and *P. minor*) were omitted from the A2 matrix following the iterative procedure outlined above. The two simultaneous analyses (A1 and A2; TreeBase study accession 11604) were performed and the trees subsequently examined to determine the degree of support for monophyletic species (A1; PSCII) and for inferring robust inter-specific relationships due to decreased missing data and the use of all available characters (A2).

**Table 2:** List of the *Pritchardia *morphological characters that were included in analysis 2

Character	Character State
1. Hastula shape	0 = rounded
	1 = triangular, apiculate
2. Degree of panicle branching	0 = two orders
	1 = three orders
3. Inflorescence length	0 = shorter than petioles
	1 = equal
	2 = longer than petioles
4. Petiole fiber density	0 = scare to moderate
	1 = abundant
5. Abaxial leaf blade folds	0 = glaucous
	1 = cottony, mealy indumentum
6. Abaxial leaf blade cover	0 = green
	1 = silvery-gray
7. Leaf blade shape	0 = nearly circular
	1 = diamond
8. Leaf blade with waxy, glaucous bloom	0 = absent
	1 = present
9. Leaf blade surface	0 = flat
	1 = nearly flat, undulate
10. Leaf tips	0 = drooping
	1 = stiff
11. Lepidia density	0 = absent
	1 = incompletely covered
	2 = completely covered
12. Rachillae tomentum	0 = glabrous
	1 = velutinous
	2 = floccose, lanate
13. Rachillae viscosity	0 = absent
	1 = present
14. Style - ovary ratio	0 = equal
	1 = style longer
	2 = style shorter
15. Outer calyx venation	0 = absent
	1 = conspicuous
	2 = present near opening with finer lines
16. Calyx indumentum	0 = glabrous
	1 = tomentose
	2 = viscous
17. Fruit ridges	0 = absent
	1 = present
18. Fruit shape	0 = globose
	1 = ellipsoid
	2 = ovoid
	3 = obovoid
	4 = oblate
19. Fruit length	0 = < 3 cm
	1 = > 3 cm

Characters states were identified from herbarium specimens at BISH, NY, PTBG, and US and derived from the most recent review of the genus [30].

### Coalescent-species-tree analysis

The coalescent species tree was inferred using *BEAST in BEAST v1.6.1 [62,103]. *BEAST infers coalescent species trees from multilocus data and has been shown to have advantages in computational speed and accuracy over similar methods when applied to rapid radiations [62] Coalescent-species-tree methods estimate each gene genealogy independently and assume that conflict between gene trees is due exclusively to incomplete lineage sorting. The sequence data from the A1 matrix was analyzed to avoid the inclusion of any potential hybrids. Each of the seven sequenced loci was unlinked to allow for variation in substitution models and the clock models for the chloroplast loci were linked to account for its presumed single hierarchical history. The analysis was run using a Yule species tree prior and the GTR+Γ model of nucleotide substitution with four rate categories. The Markov chains were run for 50 million generations and repeated 10 times to test for Markov chain Monte Carlo chain convergence and to ensure effective sample sizes (ESS) exceeded 200. Burn-in was determined in Tracer v1.5 based on ESS and parameter trajectories and was then removed in LogCombiner v1.6.1. Tree files were summarized in biopy v0.1.2 [104], the posterior was resampled, and the variance among 100 random resampled species trees was visualized in DensiTree [105]. We also estimated a single coalescent species tree in FigTree v1.3.1 by combining all tree files in LogCombiner v.1.6.1 [102]. We compared the coalescent species tree with the simultaneous analysis to determine whether accounting for incomplete lineage sorting resulted in a different topology. The coalescent species tree and the A1 and A2 topologies also allowed for testing of recent synonymy of species ([29]; *Pritchardia affinis *into *P. maideniana, P. aylmer-robinsonii *into *P. remota, P. elliptica *and *P. lanaiensis *into *P. glabrata*, and *P. limahuliensis *into *P. napaliensis*).

### Population structure analyses

To test for the presence of intermediates between Hawaiian *Pritchardia *species, five microsatellite markers [106] were amplified in 197 individuals representing all 28 of the previously recognized species. PeakScanner software was used for allele calling and FlexiBin v2 was used to bin alleles [107]. GenoDive v20b19 [108] was used to test for Hardy-Weinberg equilibrium within populations with the default settings. Using the default settings, Microchecker v.2.2.3 [109] was used to check for stutter, large-allele dropout, or evidence for null alleles based on a 99% confidence interval. A Bayesian procedure (Structure v2.3.2 [110]) was used that minimizes the deviation from Hardy-Weinberg and linkage equilibrium within each putative cluster by the fractional assignment of individual genomes to *K *populations. The admixture model was implemented with correlated allele frequencies and without the use of a priori information from populations of origin. Simulations included 10 iterations for each *K *value from *K *= 1 to 30, with a 100,000-generation burn-in and 100,000 chain length. The most probable number of genetically homogeneous groups (*K*) was determined by the Δ*K *statistical procedure [111] as implemented in Structure Harvester v0.6 [112]. Multimodality across the 10 replicate iterations of the Structure analysis was addressed by permuting 1,000 times using the greedy algorithm and averaging across membership coefficients in CLUMPP [113]; the results were graphically displayed using Distruct v1.1 [114].

### Population aggregation analysis

Mutually exclusive character states were used to test if gene flow had ceased between the sampled populations [35]. To examine whether previously recognized species were diagnosable and satisfy the PSCI, character-state differences were identified using PAA. As more populations are incorporated into PAA, each is compared to all species previously delimited. Each time a species profile is aggregated due to the inclusion of another population, the new profile is compared to all other species profiles to check if further aggregation is needed. We used PAA for the microsatellite, morphological, and sequence data independently of each other (because of differences in which terminals were sampled), and then performed PAA across all three data types to detect diagnosable groups. Missing and ambiguous data were treated as polymorphic for all states present, but these entries were not used to collapse otherwise diagnosable groups in PAA (J. I. Davis, pers. comm. 2011).

## Authors' contributions

CDB, MPS, and WLW designed the study; CDB and MJM generated the data; CDB, MPS, and WLW analyzed the data and wrote the paper. All authors read and approved the final manuscript.

## Appendix A

List of taxa sampled with taxonomic authorities, voucher information, and GenBank accession numbers for new sequences generated for this study. Fairchild Tropical Botanical Garden and National Tropical Botanic Garden are abbreviated as FTBG and NTBG respectively.

***Pritchardia affinis ***Becc.- *C. Gemmil 83 *(PTBG), FTBG DNA Bank 1850, Hawai'i; CISP4 JF904936, CISP5 JF905062, *matK *JF905351, *ndhF *JF905121, RPB2 JF905197, *trnDT *JF905269. ***P*. *affinis ***Becc.- *S. Perlman 13745 *(PTBG), FTBG DNA Bank 1851, Hawai'i; CISP4 JF904937, CISP5 JF905023, *matK *JF905352, *ndhF *JF905122, RPB2 JF905198, *trnDT *JF905270. ***P. arecina ***Becc.- *K. Wood 7991 *(PTBG), FTBG DNA Bank 1853, Maui; CISP4 JF904938, *matK *JF905353, *ndhF *JF905123, RPB2 JF905199, *trnDT *JF905271. ***P. arecina ***Becc.-*Baker 1183 *(K), Royal Botanic Gardens, Kew DNA Bank 15960, Maui; CISP4 JF904939, CISP5 JF905024, *matK *JF905354, *ndhF *JF905124, RPB2 JF905200, *trnDT *JF905272. ***P. aylmer-robinsonii ***H.St.John- FTBG Live Collection 85184C, FTBG DNA Bank 14, Ni'ihau; CISP4 JF904940, CISP5 JF905025, *matK *JF905355, *ndhF *JF905125, RPB2 JF905201, *trnDT *JF905273. ***P. aylmer-robinsonii ***H.St.John-NTBG Live Collection, Ni'ihau; CISP4 JF904941, CISP5 JF905026, *matK *JF905356, *ndhF *JF905126, RPB2 JF905202, *trnDT *JF905274. ***P. bakeri ***Hodel-Bacon Pupukea1 SN, O'ahu; CISP4 JF904942, RPB2 JF905203. ***P. bakeri ***Hodel-Bacon Pupukea2 SN, O'ahu; CISP4 JF904943, CISP5 JF905027, RPB2 JF905204. ***P. bakeri ***Hodel-Bacon Pupukea3 SN, O'ahu; CISP4 JF904944. ***P. bakeri ***Hodel-Bacon Pupukea4 SN, O'ahu; CISP4 JF904945. ***P. bakeri ***Hodel-Bacon Kuliouou3 SN, O'ahu; CISP4 JF904989, *matK *JF905402, MS JF905094, *ndhF *JF905164, *trnDT *JF905316. ***P. bakeri ***Hodel-Bacon Kuliouou5 SN, O'ahu; CISP4 JF904990, *matK *JF905403, MS JF905095, *ndhF *JF905165, *trnDT *JF905317. ***P. bakeri ***Hodel-Bacon Kuliouou8 SN, O'ahu; CISP4 JF904991, *matK *JF905404, MS JF905096, *ndhF *JF905166, RPB2 JF905244, *trnDT *JF905318. ***P. beccariana ***Rock- *J. Horn 4953 *(PTBG), FTBG DNA Bank 1863, Hawai'i; CISP4 JF904946, CISP5 JF905063, *matK *JF905357, *ndhF *JF905127, RPB2 JF905205, *trnDT *JF905275. ***P. beccariana ***Rock-*Wood 8911 *(PTBG), Hawai'i; CISP4 JF904947, CISP5 JF905028, *matK *JF905358, RPB2 JF905206, *trnDT *JF905276. ***P. elliptica ***Rock & Caum-cultivated 320 Mahana St. Lana'i City, Lana'i; CISP4 JF904948, *matK *JF905361. ***P. elliptica ***Rock & Caum-cultivated 452 Lana'i St. Lana'i City, Lana'i; CISP4 JF904949, CISP5 JF905029, *matK *JF905362, *ndhF *JF905128, RPB2 JF905207, *trnDT *JF905277. ***P. elliptica ***Rock & Caum-cultivated 712 Puulani St. Lana'i City, Lana'i; CISP4 JF904950. ***P. elliptica ***Rock & Caum-Oppenheimer SN1, Kunoa Valley, Lana'i; CISP4 JF904951, CISP5 JF905030, *matK *JF905363, *ndhF *JF905129, RPB2 JF905208, *trnDT *JF905278. ***P. elliptica ***Rock & Caum-Oppenheimer SN6, Kunoa Valley, Lana'i; CISP5 JF905031, *matK *JF905359, *ndhF *JF905130, *trnDT *JF905279. ***P. elliptica ***Rock & Caum-Oppenheimer SN7, Kunoa Valley, Lana'i; CISP4 JF904952, CISP5 JF905032, *matK *JF905364, *ndhF *JF905131, *trnDT *JF905280. ***P. elliptica ***Rock & Caum-Oppenheimer SN8, Kunoa Valley, Lana'i; CISP4 JF904953, CISP5 JF905033, *matK *JF905360, RPB2 JF905209. ***P. flynnii ***Lorence & Gemmill-*Wood 12718B *(PTBG), Kaua'i; CISP4 JF904954, *matK *JF905366, MS JF905087, RPB2 JF905210. ***P. flynnii ***Lorence & Gemmill-*Wood 12718C *(PTBG), Kaua'i; CISP4 JF904955, *matK *JF905365. ***P. flynnii ***Lorence & Gemmill-NTBG Live Collection, Kaua'i; CISP4 JF904956, CISP5 JF905034, *matK *JF905367, *ndhF *JF905132, RPB2 JF905211, *trnDT *JF905281. ***P. flynnii ***Lorence & Gemmill-*Tangalin 1476 *(PTBG), Kaua'i; CISP4 JF904957, *matK *JF905368, MS JF905097, RPB2 JF905212, *trnDT *JF905282. ***P. flynnii ***Lorence & Gemmill-*Tangalin 1478 *(PTBG), Kaua'i; CISP4 JF904958, CISP5 JF905035, *matK *JF905369, *ndhF *JF905133, RPB2 JF905213, *trnDT *JF905283. ***P. flynnii ***Lorence & Gemmill-*Tangalin 1480 *(PTBG), Kaua'i; *trnDT *JF905284. ***P. forbesiana ***Rock- *J. Horn 4948 *(FTBG), FTBG DNA Bank 1798, Maui; *matK *JF905370, *ndhF *JF905134, RPB2 JF905214, *trnDT *JF905285. ***P. forbesiana ***Rock-NTBG Live Collection, Maui; CISP4 JF904959, CISP5 JF905036, *matK *JF905371, *ndhF *JF905135, RPB2 JF905215, *trnDT *JF905286. ***P. glabrata ***Becc. & Rock-FTBG DNA Bank 824, Maui; CISP4 JF904960, CISP5 JF905037, *matK *JF905372, *ndhF *JF905136, RPB2 JF905216. ***P. glabrata ***Becc. & Rock-Oppenheimer SN1, Maui; CISP4 JF904961, CISP5 JF905038, *matK *JF905373, RPB2 JF905217, *trnDT *JF905287. ***P. glabrata ***Becc. & Rock-Oppenheimer SN4, Maui; CISP4 JF904962, CISP5 JF905039, *matK *JF905374, MS JF905098, *ndhF *JF905137, RPB2 JF905218, *trnDT *JF905288. ***P. glabrata ***Becc. & Rock-Oppenheimer SN5, Maui; CISP4 JF904963, CISP5 JF905040, *matK *JF905375, MS JF905099, *ndhF *JF905138, RPB2 JF905219, *trnDT *JF905289. ***P glabrata ***Becc. & Rock-Oppenheimer SN6, Maui; CISP4 JF904964, *matK *JF905376, *ndhF *JF905139, RPB2 JF905220, *trnDT *JF905290. ***P. hardyi ***Rock-*Trauernicht 428 *(PTBG), Kaua'i; CISP4 JF904965, *matK *JF905377, RPB2 JF905221, *trnDT *JF905291. ***P. hardyi ***Rock-*Trauernicht 429 *(PTBG), Kaua'i; *matK *JF905378, *ndhF *JF905140. ***P. hardyi ***Rock-*Trauernicht 430 *(PTBG), Kaua'i; CISP4 JF904966, *matK *JF905379, *ndhF *JF905141. ***P. hardyi ***Rock- *J. Horn 4938 *(PTBG), Kaua'i, FTBG DNA Bank 1848; CISP4 JF904967, CISP5 JF905064, *matK *JF905380, MS JF905088, *ndhF *JF905142, RPB2 JF905222, *trnDT *JF905292. ***P. hardyi ***Rock-*J. Horn 4951 *(PTBG), FTBG DNA Bank 1858, Kaua'i; CISP4 JF904968, CISP5 JF905065, *matK *JF905381, MS JF905089, *ndhF *JF905143, RPB2 JF905223, *trnDT *JF905293. ***P. hardyi ***Rock-*Tangalin 1705 *(PTBG), Kaua'i; *trnDT *JF905294. ***P. hillebrandii ***Becc.-FTBG Live Collection 2000301A, FTBG DNA Bank 646, Moloka'i; CISP4 JF904969, CISP5 JF905041, *matK *JF905382, *ndhF *JF905144, RPB2 JF905224, *trnDT *JF905295. ***P. hillebrandii ***Becc.-*S. Zona 1006 *(FTG), FTBG DNA Bank 834, Moloka'i; CISP4 JF904970, CISP5 JF905042, *matK *JF905383, *ndhF *JF905145, RPB2 JF905225, *trnDT *JF905296. ***P. kaalae ***Rock-*S. Zona 1008 *(FTG), FTBG DNA Bank 835, O'ahu; CISP4 JF904973, CISP5 JF905043, *matK *JF905386, *ndhF *JF905148, RPB2 JF905228, *trnDT *JF905299. ***P. kaalae ***Rock-*K. Wood 300 *(PTBG), FTBG DNA Bank 1833, O'ahu; CISP4 JF904971, CISP5 JF905066, *matK *JF905384, *ndhF *JF905146, RPB2 JF905226, *trnDT *JF905297. ***P. kaalae ***Rock-*S. Perlman 16710 *(PTBG), FTBG DNA Bank 1847, O'ahu; CISP4 JF904972, CISP5 JF905067, *matK *JF905385, *ndhF *JF905147, RPB2 JF905227, *trnDT *JF905298. ***P. kahukuensis ***Caum-*Kawelo SN *(BISH), O'ahu; CISP4 JF904974, CISP5 JF905044, *matK *JF905387, *ndhF *JF905149, RPB2 JF905229, *trnDT *JF905300. ***P. lanaiensis ***Becc. & Rock-Bacon 88, Lana'i; CISP4 JF904975, CISP5 JF905045, *matK *JF905388, *ndhF *JF905150, RPB2 JF905230, *trnDT *JF905301. ***P. lanaiensis ***Becc. & Rock-Bacon 126, Lana'i; CISP4 JF904976, CISP5 JF905068, *matK *JF905389, *ndhF *JF905151, RPB2 JF905231, *trnDT *JF905302. ***P. lanaiensis ***Becc. & Rock-*S. Perlman 16385 *(PTBG), FTBG DNA Bank 1845, Lana'i; CISP4 JF904977, CISP5 JF905069, *matK *JF905390, MS JF905100, *ndhF *JF905152, RPB2 JF905232, *trnDT *JF905303. ***P. lanaiensis ***Becc. & Rock-*Perlman 19968 *(PTBG), Lana'i; CISP4 JF904978, CISP5 JF905046, *matK *JF905391, *ndhF *JF905153, RPB2 JF905233, *trnDT *JF905304. ***P. lanigera ***Becc.-*K. Wood 7611 *(PTBG), FTBG DNA Bank 1846, Hawai'i; CISP4 JF904979, CISP5 JF905070, *matK *JF905392, MS JF905101, *ndhF *JF905154, RPB2 JF905234, *trnDT *JF905305. ***P. limahuliensis ***H.St.John- *J. Horn 4947 *(PTBG), FTBG DNA Bank 1831, Kaua'i; CISP4 JF904980, *matK *JF905393, MS JF905102, *ndhF *JF905155, RPB2 JF905236, *trnDT *JF905307. ***P. limahuliensis ***H.St.John-NTBG Live Collection, Kaua'i; CISP4 JF904981, CISP5 JF905071, *matK *JF905394, MS JF905103, *ndhF *JF905156, RPB2 JF905235, *trnDT *JF905308. ***P. lowreyana ***Rock ex Becc.- *J. Horn 4943 *(PTBG), FTBG DNA Bank 1794, Moloka'i; CISP4 JF904982, CISP5 JF905072, *matK *JF905395, *ndhF *JF905157, RPB2 JF905237, *trnDT *JF905309. ***P. lowreyana ***Rock ex Becc.-*Wood 9236 *(PTBG), Moloka'i; CISP4 JF904983, CISP5 JF905047, *matK *JF905396, *ndhF *JF905158, RPB2 JF905238, *trnDT *JF905310. ***P. martii ***(Gaudich.) H.Wendl.- Bakutis Waianae SN1, O'ahu; CISP4 JF904984, CISP5 JF905048, *matK *JF905397, *ndhF *JF905159, RPB2 JF905239, *trnDT *JF905311. ***P. martii ***(Gaudich.) H.Wendl.- Bakutis Waianae SN2, O'ahu; CISP4 JF904985, CISP5 JF905049, *matK *JF905398, MS JF905090, *ndhF *JF905160, RPB2 JF905240, *trnDT *JF905312. ***P. martii ***(Gaudich.) H.Wendl.- Bacon Waiava1, O'ahu; CISP4 JF904988, CISP5 JF905052, *matK *JF905401, *ndhF *JF905163, RPB2 JF905243, *trnDT *JF905315. ***P. martii ***(Gaudich.) H.Wendl.- Bacon Waiava7, O'ahu; CISP4 JF904986, CISP5 JF905050, *matK *JF905399, MS JF905104, *ndhF *JF905161, RPB2 JF905241, *trnDT *JF905313. ***P. martii ***(Gaudich.) H.Wendl.- Bacon Waiava15, O'ahu; CISP4 JF904987, CISP5 JF905051, *matK *JF905400, *ndhF *JF905162, RPB2 JF905242, *trnDT *JF905314. ***P. martii ***(Gaudich.) H.Wendl.- *J. Horn 4937 *(PTBG), FTBG DNA Bank 1855, O'ahu; CISP4 JF904992, CISP5 JF905073, *matK *JF905405, *ndhF *JF905167, RPB2 JF905245, *trnDT *JF905319. ***P. martii ***(Gaudich.) H.Wendl.- *J. Horn 4954 *(PTBG), FTBG DNA Bank 1859, O'ahu; CISP4 JF904993, CISP5 JF905074, *matK *JF905406, *ndhF *JF905168, RPB2 JF905246, *trnDT *JF905320. ***P. martii ***(Gaudich.) H.Wendl.-NTBG live collection, O'ahu; CISP4 JF904994, CISP5 JF905053, *matK *JF905406, *ndhF *JF905169. ***P. minor ***Becc.-*Trauernicht 432 *(PTBG), Kaua'i; *matK *JF905408. ***P. minor ***Becc.-*Trauernicht 434 *(PTBG), Kaua'i; CISP4 JF904995, *matK *JF905409, *ndhF *JF905170. ***P. minor ***Becc.-*Trauernicht 435 *(PTBG), Kaua'i; CISP4 JF904996, *matK *JF905410, MS JF905105, *ndhF *JF905171, *trnDT *JF905321. ***P. minor ***Becc.-*J. Horn 4946 *(PTBG), FTBG DNA Bank 1797, Kaua'i; CISP4 JF904997, CISP5 JF905075, *matK *JF905411, *ndhF *JF905172, RPB2 JF905247, *trnDT *JF905322. ***P. minor ***Becc.- *S. Zona 1033 *(FTG), FTBG DNA Bank 845, Kaua'i; CISP4 JF904998, CISP5 JF905054, *matK *JF905412, *ndhF *JF905173, RPB2 JF905248, *trnDT *JF905323. ***P. minor ***Becc.- -*Tangalin 1708 *(PTBG), Kaua'i; *trnDT *JF905324. ***P. mitiaroana ***J.Drans. & Y.Ehrh.- *S. Perlman 19346 *(PTBG), FTBG DNA Bank 1857, Cook Islands; CISP4 JF904999, CISP5 JF905076, *matK *JF905413, MS JF905091, *ndhF *JF905174, RPB2 JF905249, *trnDT *JF905325. ***P. mitiaroana ***J.Drans. & Y.Ehrh.-*Perlman 19346 *(PTBG), Cook Islands; CISP4 JF905000. ***P***. cf. ***mitiaroana ***J.Drans. & Y.Ehrh.-Meyer SN 'pericularum', French Polynesia; CISP4 JF905006, CISP5 JF905057, *matK *JF905419, MS JF905108, *ndhF *JF905181, RPB2 JF905254, *trnDT *JF905332. **P**. cf. ***mitiaroana ***J.Drans. & Y.Ehrh.-Meyer SN 'vuylstekeana', French Polynesia; CISP4 JF905019, CISP5 JF905085, *matK *JF905434, MS JF905118, *ndhF *JF905193, RPB2 JF905265, *trnDT *JF905346. ***P. munroi ***Rock- *J. Horn 4942 *(PTBG), FTBG DNA Bank 1832, Moloka'i; CISP4 JF905001, CISP5 JF905077, *matK *JF905414, *ndhF *JF905175, RPB2 JF905250, *trnDT *JF905326. ***P. munroi ***Rock- *S. Zona 1036 *(FTG), FTBG DNA Bank 841, Moloka'i; CISP4 JF905002, CISP5 JF905055, *matK *JF905415, *ndhF *JF905176, RPB2 JF905251, *trnDT *JF905327. ***P. napaliensis ***H.St.John- *S. Perlman 11297 *(PTBG), FTBG DNA Bank 1860, Kaua'i; CISP4 JF905003, CISP5 JF905078, *matK *JF905416, MS JF905106, *ndhF *JF905177, RPB2 JF905268, *trnDT *JF905328. ***P. napaliensis ***H.St.John-*Wood 9087 *(PTBG), Kaua'i; CISP4 JF905004, CISP5 JF905056, *matK *JF905417, MS JF905092, *ndhF *JF905178, *trnDT *JF905329. ***P. pacifica ***Seem. & H.Wendl.- FTBG Live Collection 93691D, FTBG DNA Bank 18, Fiji; CISP4 JF905005, CISP5 JF905079, *ndhF *JF905179, RPB2 JF905252, *trnDT *JF905330. ***P. pacifica ***Seem. & H.Wendl.-*J. Horn 4952 *(PTBG), FTBG DNA Bank 1861, Fiji; CISP5 JF905080, *matK *JF905418, MS JF905107, *ndhF *JF905180, RPB2 JF905253, *trnDT *JF905331. ***P. perlmanii ***Gemmill-*Wood 7331 *(PTBG), Kaua'i; CISP4 JF905007, *matK *JF905421, MS JF905109, *ndhF *JF905183, *trnDT *JF905333. ***P. perlmanii ***Gemmill-*Wood 8091 *(PTBG), Kaua'i; CISP4 JF905008, CISP5 JF905058, *matK *JF905422, MS JF905110, *ndhF *JF905184, RPB2 JF905255, *trnDT *JF905334. ***P. perlmanii ***Gemmill-NTBG Live Collection, Kaua'i; *matK *JF905420, MS JF905111, *ndhF *JF905182, *trnDT *JF905335. ***P. remota ***(Kuntze) Becc.- *J. Horn 4955 *(PTBG), FTBG DNA Bank 1844, Nihoa; CISP4 JF905009, CISP5 JF905081, *matK *JF905423, *ndhF *JF905185, RPB2 JF905256, *trnDT *JF905336. ***P. remota ***(Kuntze) Becc.-*J. Horn 4936 *(PTBG), FTBG DNA Bank 1865, Nihoa; CISP4 JF905010, CISP5 JF905082, *matK *JF905424, *ndhF *JF905186, RPB2 JF905257, *trnDT *JF905337. ***P. remota ***(Kuntze) Becc.-Montgomery Botanical Center Live Collection 29, Nihoa; CISP4 JF905011, *matK *JF905425, MS JF905112, RPB2 JF905258, *trnDT *JF905338. ***P. schattaueri ***Hodel-*J. Horn 4939 *(PTBG), FTBG DNA Bank 1843, Hawai'i; CISP4 JF905012, CISP5 JF905083, *matK *JF905426, MS JF905113, *ndhF *JF905187, RPB2 JF905259, *trnDT *JF905339. ***P. schattaueri ***Hodel- *S. Zona 1001 *(FTG), FTBG DNA Bank 839, Hawai'i; CISP4 JF905013, CISP5 JF905059, *matK *JF905427, MS JF905114, *ndhF *JF905188, RPB2 JF905260, *trnDT *JF905340. ***P. thurstonii ***F.Muell. & Drude-NTBG Live Collection, Fiji; CISP4 JF905014, CISP5 JF905060, *matK *JF905428, MS JF905115, *ndhF *JF905189, RPB2 JF905261, *trnDT *JF905341. ***P. viscosa ***Rock- *J. Horn 4943 *(PTBG), FTBG DNA Bank 1795, Kaua'i; CISP4 JF905015, CISP5 JF905084, *matK *JF905429, *ndhF *JF905190, RPB2 JF905262, *trnDT *JF905342. *matK *JF905430, *ndhF *JF905191. ***P. viscosa ***Rock-*Tangalin 1693 *(PTBG), Kaua'i; CISP4 JF905016, *matK *JF905431, MS JF905116, RPB2 JF905263, *trnDT *JF905343. ***P. viscosa ***Rock-*Tangalin 1694 *(PTBG), Kaua'i; CISP4 JF905017, *matK *JF905432, MS JF905117, RPB2 JF905264, *trnDT *JF905344. ***P. viscosa ***Rock-*Perlman 16679A *(PTBG), Kaua'i; CISP4 JF905018, *matK *JF905433, MS JF905093, *ndhF *JF905192, *trnDT *JF905345. ***P. waialealeana ***Read-*Trauernicht 423 *(PTBG), Kaua'i; *matK *JF905436, *trnDT *JF905347. ***P. waialealeana ***Read-*Lorence 8446 *(PTBG), Kaua'i; CISP4 JF905021, *matK *JF905435, *ndhF *JF905194, *trnDT *JF905348. ***P. waialealeana ***Read- *J. Horn 4950 *(PTBG), FTBG DNA Bank 1863, Kaua'i; CISP4 JF905020, CISP5 JF905086, *matK *JF905437, MS JF905119, *ndhF *JF905195, RPB2 JF905266, *trnDT *JF905349. ***P. waialealeana ***Read-NTBG Live Collection, Kaua'i; CISP4 JF905022, CISP5 JF905061, *matK *JF905438, MS JF905120, *ndhF *JF905196, RPB2 JF905267, *trnDT *JF905350.

## Appendix B

## Supplementary Material

Additional file 1 **Figure S1**. Parsimony strict consensus trees of all the sequence data summarized to show only the inter-generic relationships and *Pritchardia *from different island chains. Parsimony jackknife support values above, and likelihood bootstrap values below each branch of each gene individually, the plastid partition, and the simultaneous analysis. Click here for file

Additional file 2 Table S1. Mutually exclusive character states were used to test if gene flow had ceased between the sampled populations using population aggregation analysis for each of the three datasets listed in columns with spaces between each of the independent lineages. In the sequence dataset, terminals with missing data for diagnostic characters were arbitrarily assigned to a single group rather than collapsing the otherwise diagnosable groups and are indicated with *. Click here for file

Additional file 3 Figure S2. Parsimony simultaneous analysis and strict consensus tree of all the 105 terminals sampled for nucleotide data with parsimony jackknife values shown. Click here for file

Additional file 4 Figure S3. The individual nuclear gene trees estimated for Pritchardia species delimitation as shown in the parsimony strict consensus with parsimony jackknife values above and likelihood bootstrap values below each branch. Click here for file

Additional file 5 Figure S4. The individual plastid gene trees and the plastid simultaneous-analysis estimated for Pritchardia species delimitation with jackknife branch support values above and bootstrap values below each branch. Click here for file
