# Supplementary material for: Evaluating multiple criteria for species delimitation: an empirical example using Hawaiian palms (Arecaceae: Pritchardia)
Source: BMC Evol Biol. 2012 Feb 22;12:23. doi: 10.1186/1471-2148-12-23 (PMC3356231; doi:10.1186/1471-2148-12-23)
Supplement: Additional file 1 — Figure S1. Parsimony strict consensus trees of all the sequence data summarized to show only the inter-generic relationships and Pritchardia from different island chains. Parsimony jackknife support values above, and likelihood bootstrap values below each branch of each gene individually, the plastid partition, and the simultaneous analysis. [file 1471-2148-12-23-S1.PDF]

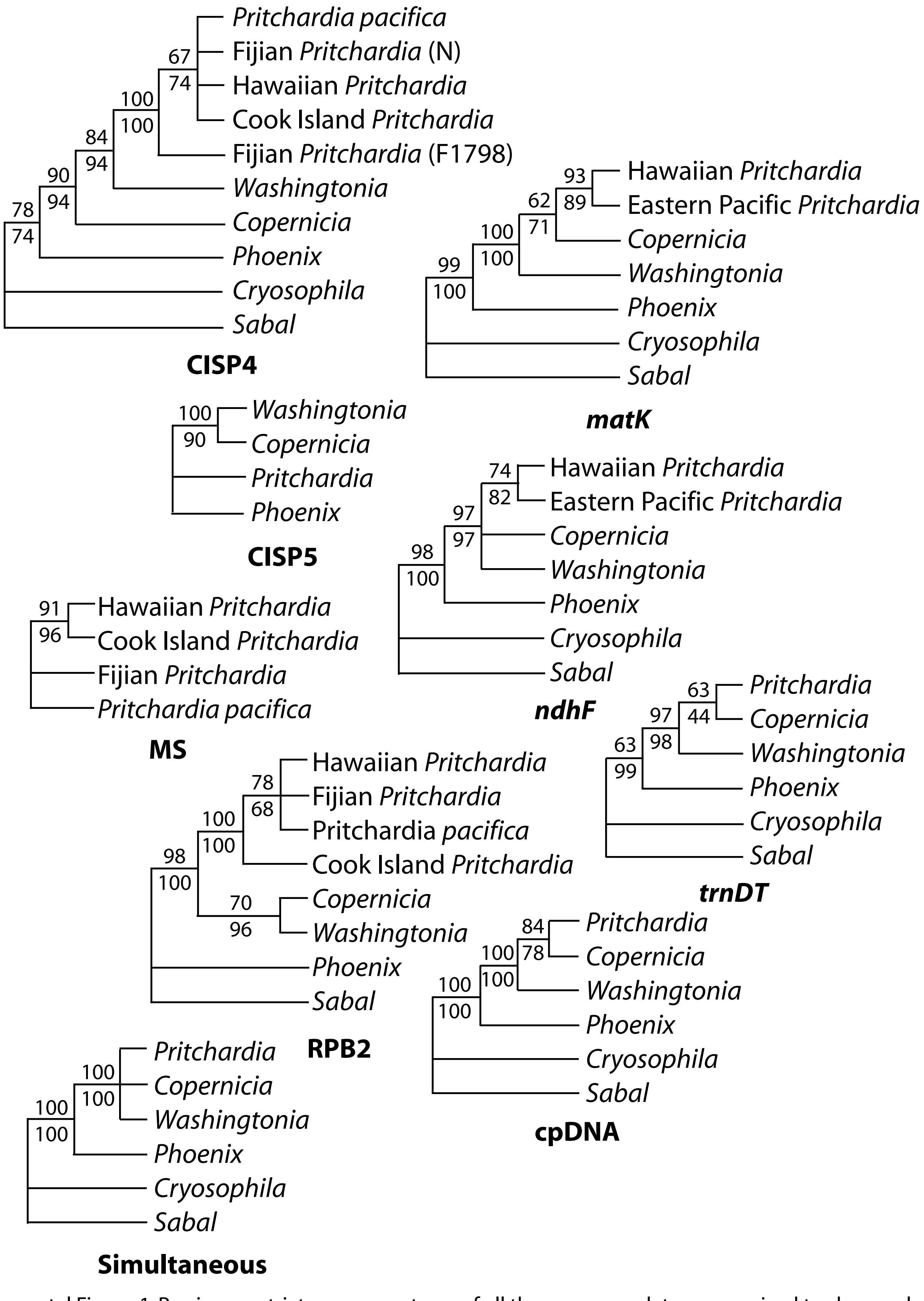

Supplemental Figure 1. Parsimony strict consensus trees of all the sequence data summarized to show only the inter-generic relationships and Pritchardia from different island chains. Parsimony jackknife support values above, and likelihood bootstrap values below each branch of each gene individually, the plastid partition, and the simultaneous analysis.
