# Supplementary material for: Evaluating multiple criteria for species delimitation: an empirical example using Hawaiian palms (Arecaceae: Pritchardia)
Source: BMC Evol Biol. 2012 Feb 22;12:23. doi: 10.1186/1471-2148-12-23 (PMC3356231; doi:10.1186/1471-2148-12-23)
Supplement: Additional file 2 — Table S1. Mutually exclusive character states were used to test if gene flow had ceased between the sampled populations using population aggregation analysis for each of the three datasets listed in columns with spaces between each of the independent lineages. In the sequence dataset, terminals with missing data for diagnostic characters were arbitrarily assigned to a single group rather than collapsing the otherwise diagnosable groups and are indicated with *. [file 1471-2148-12-23-S2.PDF]

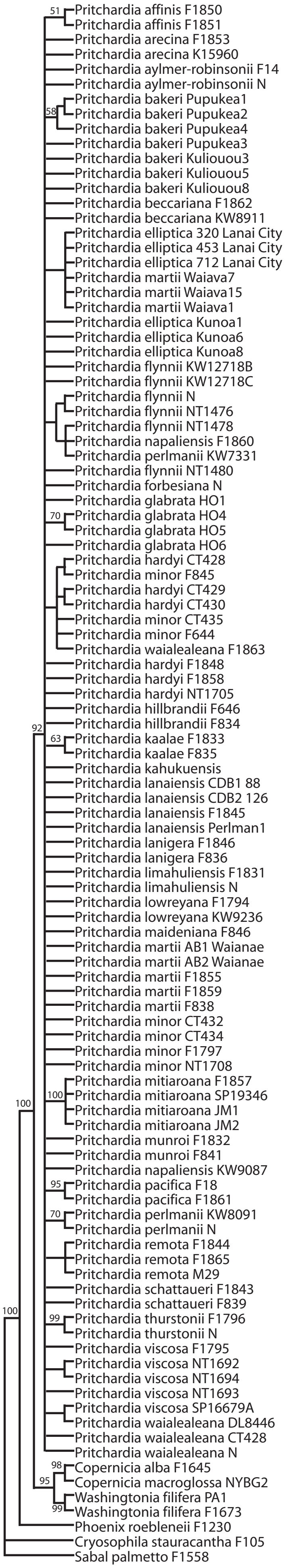

Supplemental Figure 2. Parsimony simultaneous analysis and strict consensus tree of all the 105 terminals sampled for nucleotide data with parsimony jackknife values shown.
