# Supplementary material for: Evaluating multiple criteria for species delimitation: an empirical example using Hawaiian palms (Arecaceae: Pritchardia)
Source: BMC Evol Biol. 2012 Feb 22;12:23. doi: 10.1186/1471-2148-12-23 (PMC3356231; doi:10.1186/1471-2148-12-23)
Supplement: Additional file 3 — Figure S2. Parsimony simultaneous analysis and strict consensus tree of all the 105 terminals sampled for nucleotide data with parsimony jackknife values shown. [file 1471-2148-12-23-S3.PDF]

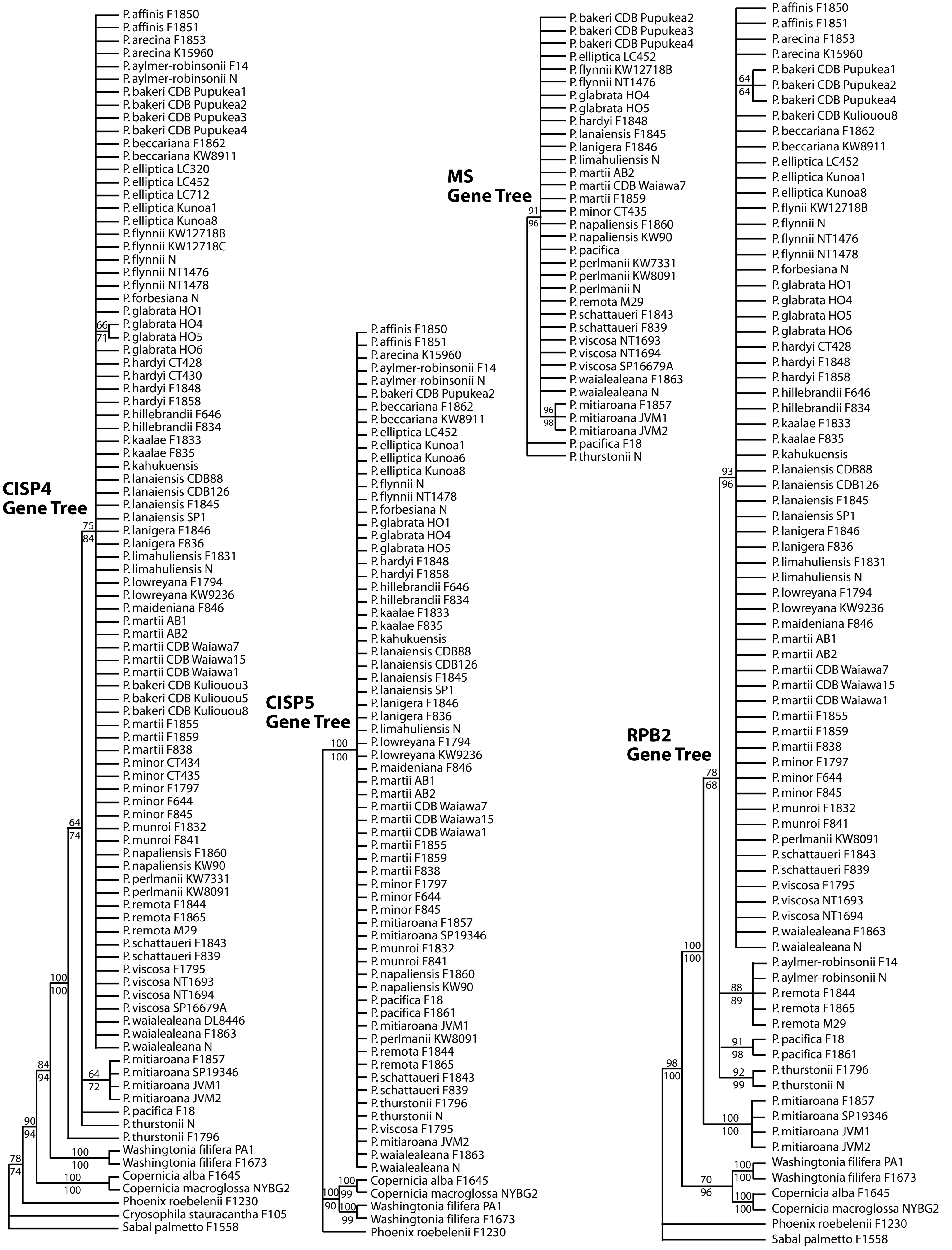

Supplemental Figure 3. The individual nuclear gene trees estimated for *Pritchardia* species delimitation as shown in the parsimony strict consensus with parsimony jackknife values above and likelihood bootstrap values below each branch.
