# Supplementary material for: Evaluating multiple criteria for species delimitation: an empirical example using Hawaiian palms (Arecaceae: Pritchardia)
Source: BMC Evol Biol. 2012 Feb 22;12:23. doi: 10.1186/1471-2148-12-23 (PMC3356231; doi:10.1186/1471-2148-12-23)
Supplement: Additional file 4 — Figure S3. The individual nuclear gene trees estimated for Pritchardia species delimitation as shown in the parsimony strict consensus with parsimony jackknife values above and likelihood bootstrap values below each branch. [file 1471-2148-12-23-S4.PDF]

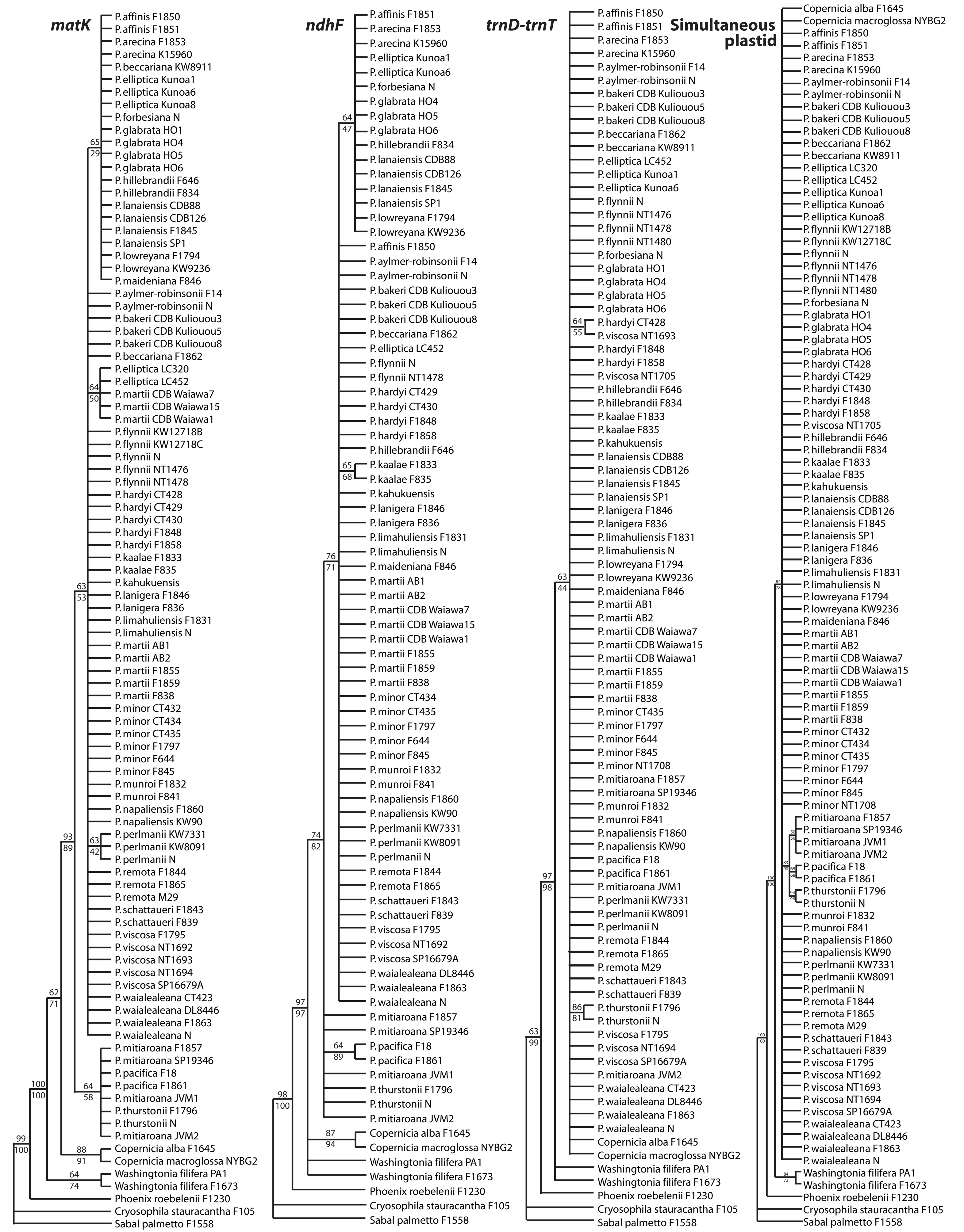

Supplemental Figure 4. The individual plastid gene trees and the plastid simultaneous-analysis estimated for *Pritchardia* species delimitation with jackknife branch support values above and bootstrap values below each branch.
