# Supplementary material for: Evaluating multiple criteria for species delimitation: an empirical example using Hawaiian palms (Arecaceae: Pritchardia)
Source: BMC Evol Biol. 2012 Feb 22;12:23. doi: 10.1186/1471-2148-12-23 (PMC3356231; doi:10.1186/1471-2148-12-23)
Supplement: Additional file 5 — Figure S4. The individual plastid gene trees and the plastid simultaneous-analysis estimated for Pritchardia species delimitation with jackknife branch support values above and bootstrap values below each branch. [file 1471-2148-12-23-S5.PDF]

Supplemental Table 1. Mutually exclusive character states were used to test if gene flow had ceased between the sampled populations using population aggregation analysis for each of the three datasets listed in columns with spaces between each of the independent lineages. In the sequence dataset, terminals with missing data for diagnostic characters were arbitrarily assigned to a single group rather than collapsing the otherwise diagnosable groups and are indicated with *.

**Microsatellites**	**Sequences**	**Morphology**
arecina (24)	affinis (FTBG 1850)	affinis
arecina (NTBG 158)		maideniana
arecina (245)	affinis (FTBG 1851)	
arecina (246)		arecina
arecina (247)	arecina (RGBK 15960)	
bakeri (Kul 2_1)	lowreyana (KW 9236)	aylmer-robinsonii
bakeri (Kul 2_2)	lanaiensis (CB 88, CB 126)	remota
bakeri (49)		thurstonii
bakeri (Kul 2_4)	aylmer-robinsonii (FTBG 14, NTBG)	
bakeri (50)		bakeri
bakeri (Kul 2_7)	bakeri (Pupu 1, 2, 4)	flynii
bakeri (51)		martii
bakeri (Kul 2_26)	kahukuensis	minor
bakeri (Pupu 1)	bakeri (Kul 3, 5, 8)	kahukuensis
bakeri (Pupu 2)	martii (Waianae 44, 45, FTBG 838)	perlmanii
bakeri (Pupu 3K)		
bakeri (Pupu 4)	elliptica (320, 452)	forbesiana
bakeri (Pupu 5K)	martii (Waiawa 1, 7, 15)	schattaueri
bakeri (Pupu 6)		
bakeri (Pupu 7)	beccariana (KW 8911)	gordonii
bakeri (Pupu 8)	elliptica (Kunoa 1)	
bakeri (Pupu 9)		hardyi
bakeri (Pupu 10)	flynnii (NTBG)	
bakeri (Pupu 11)		hillebrandii
bakeri (Pupu 12)	flynnii (NT 1476, NT 1478)	
bakeri (Pupu 13)		glabrata
bakeri (Pupu 14)	glabrata (HO 1, 4, 5, 6)	kaalae
bakeri (Pupu 15K)		lanaiensis
bakeri (Pupu 16)	hardyi (CT 428, CT429)	lanigera
bakeri (Pupu 17)		limahuliensis
bakeri (Pupu 18)	kaalae (FTBG 835, FTBG 1833)	lowreyana
bakeri (Pupu 19)		munroi
bakeri (Pupu 20K)	lanaiensis (FTBG 1845)	napaliensis
bakeri (Pupu 21K)		woodii
bakeri (Pupu 22K)	lanaiensis (SP 1)	beccariana

bakeri (Pupu 23)		elliptica
bakeri (Pupu 24)	limahuliensis (NTBG)	mitiaroana
bakeri (Pupu 25)		
bakeri (Pupu 26)	lowreyana (FTBG 1794)	pacifica
bakeri (Pupu 27)		
bakeri (Pupu 28)	maideniana (FTBG 846)	viscosa
beccariana 22		
beccariana 25	mitiaroana (FTBG 1857)	waialealeana
beccariana (NTBG 160)		
beccariana (NTBG 200)	mitiaroana (SP 19346)	
beccariana (KW 8911)		
beccariana (NTBG 227)	napaliensis (FTBG 1860)	
flynnii (33)		
flynnii (34)	pacifica (FTBG 18)	
flynnii (NTBG 162)		
flynnii (NT 1476)	pacifica (FTBG 1861)	
flynnii (NT 1478)		
forbesiana (FTBG 1798)	pericularum (JM 1)	
elliptica (29)		
elliptica (30)	perlmanii (KW 7331)	
elliptica (31)		
elliptica (32)	perlmanii (KW 8091, NTBG)	
elliptica (26)		
elliptica (27)	remota (FTBG 1844, FTBG 1865, MBC 29)	
elliptica (28)		
elliptica (NTBG 161)	schattaueri (FTBG 1843)	
glabrata (35)		
glabrata (36)	thurstonii (FTBG 1796)	
glabrata (37)		
glabrata (38)	thurstonii (NTBG)	
lanaiensis (40)		
lanaiensis (41)	vuylstekeana (JM 2)	
lanaiensis (42)		
lanaiensis (NTBG 167)	waialealeana (FTBG 1863)	
hardyi (NTBG 164)		
hardyi (CT 429)	flynnii (KW 12718B)*	
hardyi (CT 428)	lanigera (FTBG 1846)*	
hardyi (CT 430)	limahuliensis (FTBG 1831)*	
hardyi (MC 9)	minor (CT4 35, FTBG 845)*	
hillabrandii (NTBG 165)	napaliensis (KW 9081)*	
hillebrandii (KW 8043)	viscosa (NT 1692)*	
hillebrandii (KW 3838)		
hillebrandii (KW 9196)		

hillebrandii (KW 9200)
hillebrandii (KW 9198)
hillebrandii (KW 9206)
kaalae (NTBG 166)
kaalae (LW 1)
kaalae (MMR A89)
kaalae (MMR B3)
kahukuensis (39)
lanigera (NTBG 168)
lanigera (KW 7611)
lanigera (KW 7812)
lanigera (FTBG 1846)
cf lanigera (235)
lowreyana (KW 9236)
lowreyana (NTBG 170)
lowreyana (KW 9236)
lowreyana (KW 7516)
affinis (NTBG 157)
affinis (PTBG 890115)
affinis (PTBG 830115)
affinis (FTBG 1851)
martii (Waianae 44)
martii (Waianae 45)
martii (NTBG 155)
martii (Ewa 1)
martii (Ewa 2)
martii (Ewa 10K)
martii (Ewa 12)
martii (Ewa 13)
martii (Ewa 15)
martii (Ewa 16)
martii (Ewa 18)
martii (Ewa 20)
martii (Ewa 22)
martii (Ewa 23)
martii (Ewa 24)
martii (Ewa 25)
martii (Ewa 26)
martii (Ewa 27)
martii (Ewa 29)
martii (Ewa 34)
martii (Ewa 35K)
martii (Ewa 36)

martii (Ewa 37)

martii (Ewa 38)

martii (Ewa 39)

martii (Ewa 40)

martii (Ewa 41)

martii (Ewa 42)

martii (Ewa 43)

martii (Ewa 44)

martii (Ewa 45K)

martii (Ewa 46)

martii (Ewa 47)

martii (Waiawa 2)

martii (Waiawa 3)

martii (Waiawa 4)

martii (Waiawa 48)

martii (Waiawa 46)

martii (Waiawa 8)

martii (Waiawa 9)

martii (Waiawa 10)

martii (Waiawa 11)

martii (Waiawa 13)

martii (Waiawa 47)

martii (Waiawa 16)

martii (Waiawa 18)

martii (Waiawa 19)

martii (Waiawa 20)

martii (Waiawa 22)

martii (Waiawa 77)

martii (Waiawa 2)

martii (Waiawa 5)

martii (Waiawa 8)

martii (Waiawa 9)

martii (Waiawa 10)

martii (Waiawa 12)

martii (Waiawa 14)

martii (Waiawa 15)

martii (Waiawa 16)

martii (Waiawa 21)

martii (Waiawa 23)

martii (Waiawa 24)

martii (Waiawa 25)

martii (Waiawa 26)

martii (Waiawa 27)

martii (Waiawa 28)

minor (NTBG 18)

minor (CT 435)

minor (CT 432)

minor (CT 434)

mitiaroana (52)

mitiaroana (SP 19346A)

mitiaroana (SP 19346B)

mitiaroana (FTBG 1857)

pericularum (57)

vuylstekeana (59)

munroi (53)

munroi (NTBG 172)

munroi (SP 18256)

munroi (NTBG 244)

limahuliensis (NTBG 16)

limahuliensis (PTBG 980806)

limahuliensis (PTBG 980811)

limahuliensis (PTBG 980805)

napaliensis (54)

napaliensis (NTBG 173)

napaliensis (PTBG 930109)

pacifica (CB 219)

pacifica (CB 223)

pacifica (NTBG 238)

pacifica (NTBG 240)

pacfica (NTBG 250)

perlmanii (55)

perlmanii (56)

perlmanii (NTBG 174)

perlmanii (KW 1545)

perlmanii (NTBG 249)

perlmanii (NTBG 239)

aylmer-robinsonii (NTBG 159)

aylmer-robinsonii (197)

remota (NTBG 175)

remota (NTBG 198)

remota (MBC 29)

remota (FTBG 1865)

schattaueri (NTBG 176)

schattaueri (PTBG 920052)

schattaueri (FTBG 1843)

schattaueri NTBG (251)

schattaueri (SZ 1001A)
thurstonii (NTBG 177)
thurstonii (CB 50)
thurstonii (CB 20)
thurstonii (NTBG 242)
viscosa (58)
viscosa (NTBG 178)
viscosa (NT 1693)
viscosa (NT 1694)
viscosa (NT 1692)
viscosa (MC 2)
waialealeana (60)
waialealeana (NTBG 179)
waialealeana (CT 423)
waialealeana (241)

forbesiana (61)
forbesiana (NTBG 163)
forbesiana (PTBG 950275)
forbesiana (248)

napaliensis (NTBG 173)

schattaueri (SZ 1001B)
